# Care of cancer patients at the end of life in a German university hospital: A retrospective observational study from 2014

**DOI:** 10.1371/journal.pone.0175124

**Published:** 2017-04-06

**Authors:** Burkhard Dasch, Helen Kalies, Berend Feddersen, Caecilie Ruderer, Wolfgang Hiddemann, Claudia Bausewein

**Affiliations:** 1 Department of Palliative Medicine, Munich University Hospital, Ludwig Maximilian University of Munich, Munich, Germany; 2 Specialized Palliative Home Care, Districts of Berchtesgaden and Traunstein, Germany; 3 Department of Internal Medicine III, Munich University Hospital, Ludwig Maximilian University of Munich, Munich, Germany; Seoul National University College of Pharmacy, REPUBLIC OF KOREA

## Abstract

**Background:**

Cancer care including aggressive treatment procedures during the last phase of life in patients with incurable cancer has increasingly come under scrutiny, while integrating specialist palliative care at an early stage is regarded as indication for high quality end-of-life patient care.

**Aim:**

To describe the demographic and clinical characteristics and the medical care provided at the end of life of cancer patients who died in a German university hospital.

**Methods:**

Retrospective cross-sectional study on the basis of anonymized hospital data for cancer patients who died in the Munich University Hospital in 2014. Descriptive analysis and multivariate logistic regression analyses for factors influencing the administration of aggressive treatment procedures at the end of life.

**Results:**

Overall, 532 cancer patients died. Mean age was 66.8 years, 58.5% were men. 110/532 (20.7%) decedents had hematologic malignancies and 422/532 (79.3%) a solid tumor. Patients underwent the following medical interventions in the last 7/30 days: chemotherapy (7.7%/38.3%), radiotherapy (2.6%/6.4%), resuscitation (8.5%/10.5%), surgery (15.2%/31.0%), renal replacement therapy (12.0%/16.9%), blood transfusions (21.2%/39.5%), CT scan (33.8%/60.9%). In comparison to patients with solid tumors, patients with hematologic malignancies were more likely to die in intensive care (25.4% vs. 49.1%; *p* = 0.001), and were also more likely to receive blood transfusions (OR 2.21; 95% CI, 1.36 to 3.58; *p* = 0.001) and renal replacement therapy (OR 2.65; 95% CI, 1.49 to 4.70; *p* = 0.001) in the last 7 days of life. Contact with the hospital palliative care team had been initiated in 161/532 patients (30.3%). In 87/161 cases (54.0%), the contact was initiated within the last week of the patient’s life.

**Conclusions:**

Overambitious treatments are still reality at the end of life in cancer patients in hospital but patients with solid tumors and hematologic malignancies have to be differentiated. More efforts are necessary for the timely inclusion of specialist palliative care.

## Introduction

Cancer is the second most frequent cause of death worldwide, after cardiovascular diseases. According to estimates published by the International Agency for Research on Cancer (IARC), there were 14.9 million new cases of cancer and 8.2 million deaths from cancer in 2012 [1].

Patients with advanced stages of cancer have a very high probability of spending the last phase of their life in hospital and dying there [2–5]. Although patient surveys show that the home environment is the preferred place of death, in reality hospital is by far the most frequent location [6–8].

Physicians who treat patients in the last phase of their lives have to achieve a challenging balance in relation to the appropriateness of the decisions they take with regard to medical treatment. When is aggressive therapy justified towards the end of life, and when should attention turn towards palliative care? It is not an easy decision, and it is also made more difficult by patients sometimes requesting intensive treatment measures even when the prognosis is extremely limited [9,10].

The issue of what represents good care for cancer patients at the end of life is attracting increasing interest in scientific research. Earle et al. approached the question by conducting a literature review, focus group involvement, and expert discussions to define specific clinical quality indicators for overtreatment, incorrect treatment, and undertreatment [11]. The indicators identified were: 1) receiving chemotherapy in the last 14 days of life and/or start of chemotherapy during the last 30 days of life (overtreatment); 2) more than one emergency hospital admission and/or intensive-care unit admission during the last month of life (incorrect treatment); 3) involvement of hospice and/or specialized palliative services less than 3 days before death (undertreatment). In addition, benchmarking standards for aggressive treatment at the end of life were defined—e.g., that cytostatic therapy should be administered within the last 14 days of life in fewer than 10% of the patients treated [12].

In accordance with these criteria, studies have shown that the intensiveness of medical treatments administered at the end of life has increased in recent years [13,14]. For example, using data from 215,484 patients with statutory health insurance in the United States who were over 65 years of age and died between 1991 and 2000, it has been shown that the proportion of those who had still been receiving chemotherapy 14 days before they died rose from 9.7% in 1993 to 11.6% in 1999 [13]. Ho et al. [14] showed that in 227,161 adult cancer patients who died in Ontario between 1993 and 2004, the percentage of patients who were still receiving chemotherapy within the last 14 days of their lives increased moderately from 2.02% to 2.88%. Increasing trends were also observed with regard to multiple emergency admissions (8.60% vs. 10.53%) and admission to an intensive care unit (3.06% vs. 5.39%) during the last month of life.

Following the publication of the often-cited Temel study [15], clinical quality indicators have gained importance internationally. Jennifer S. Temel and colleagues showed that including early palliative care in the treatment of patients with metastatic non–small cell lung carcinoma (NSCLC) not only improves their quality of life and reduces anxiety and depression, but in addition leads to longer survival. Patients receiving additional palliative care received significantly fewer overambitious oncological treatments than other patients (33% vs. 54%; *p* = 0.05).

On the basis of these results and those of other randomized studies on the topic of “early integration of palliative care,” an expert committee in the American Society of Clinical Oncology (ASCO) recommended that all patients with metastatic cancer and/or a high symptom burden should be offered a combination of standard oncologic therapy and palliative care [16]. Similar recommendations have been issued by the European Society of Medical Oncology (ESMO) [17]. The following three statements regarding the time point at which palliative care should be included were published in an evidence and consensus based German guideline on palliative medicine for patients with incurable cancer [18]: 1) all patients with cancer should receive information about palliative care, independent of the disease stage; 2) following diagnosis of incurable cancer, all patients should be offered palliative care, independently of whether tumor-specific treatment is being administered; 3) specialist palliative care should be integrated into oncological decision-making processes—e.g., through involvement in interdisciplinary tumor conferences.

In Germany, 47% of the population dies in hospital and one in four citizens dies of cancer. However, little is known about the care provided during the last phase of their lives for cancer patients who die in hospital. The aim of this study was to describe the demographic and clinical characteristics and medical care of cancer patients who died at a university hospital in Germany.

## Materials and methods

### Study design

This retrospective cross-sectional study is based on hospital data for patients who died in the Munich University Hospital of Ludwig Maximilian University, Germany, between January 1^st^ 2014 and December 31^st^ 2014. With more than 2,200 beds, Munich University Hospital—with its 45 divisions, institutes, and departments in every field of medicine—is the second largest maximum-care hospital in Germany.

### Data source

Anonymized patient data from the hospital information system were analyzed. We received a complete data set including information about age, gender, date of death, diagnosis, operations, procedures, and general in-hospital medical interventions, as well as the date of documentation. In addition, information about the place of death within the hospital was evaluated.

### Study population

The study population consisted of a subgroup of all patients who died at the university hospital during the year 2014 (n = 1,222) and was exclusively related to deceased patients with a diagnosis of cancer (C00–C96) in accordance with version 10 of the International Classification of Diseases (ICD-10). In addition, cancer patients were divided into two sub-groups: a) patients with a solid tumor (C00-C80) and b) patients with malignant neoplasms of lymphoid, hematopoietic and related tissue (C81-C96). This subdivision was made to consider cancer patients more differentiated regarding their tumor-specific treatment options.

### Operations, procedures, and medical interventions

In order to investigate whether overtreatment, incorrect treatment, or undertreatment occurred in cancer patients at the end of their lives, code numbers from the *Operationen- und Prozedurenschlüssel* (OPS)—the German version of the International Classification of Procedures in Medicine (2013 version)—were extracted from the hospital information system for the periods of the last 7 days and 30 days before the patient’s death and analyzed. Chemotherapy was assumed to be present if OPS codes 8–541, 8–542, 8–543, 8–544, 8–546, 8–547, 8–549, or 6–00 in combination with code 8–54, were documented. The term “chemotherapy” included all classic types of cytostatic agent, hormones and hormone antagonists used to treat cancer, immunotherapeutic agents used to treat cancer (monoclonal antibodies, cytokines), and what are known as “small molecules” (e.g., tyrosine kinase inhibitors). The following additional medical OPS codes were also analyzed: radiotherapy (8–520 to 8–526, 8–52a to 8–52d), resuscitation (8–771), any operations (5–01 to 5–99), tracheotomy (5–312), percutaneous endoscopic gastrostomy (5–431.2), thoracentesis (8–152.2), ascites puncture (8–153), extracorporeal membrane oxygenation (ECMO) (8–852.0 to 8–852.0a), renal replacement therapy (8–853 to 8–857, 8–85a), blood cell transfusion (8–800 to 8–805), erythrocyte transfusion (8–800.c), platelet transfusion (8–800.6, 8–800.d, 8–800.f, 8–800.g, 8.800.h), blood plasma transfusion (8–810 to 8–812), tracheo-bronchoscopy (1–620), upper gastrointestinal endoscopy (1–630 to 1–638), computed tomography (CT) (3–20 to 3–24), and magnetic resonance imaging (MRI) (3–80 to 3–84).

### Palliative care service

The hospital palliative care team in the Department of Palliative Medicine at Munich University Hospital is a multiprofessional team that provides specialist palliative care for patients with advanced disease and in the terminal phase in every ward in the hospital. This includes continuous palliative consultation and collaborative treatment in patients with complex symptoms and needs. The team consists of physicians, palliative care nurses, social workers, psychologists, a respiratory therapist and a permanent member of the hospital’s pastoral care service. Via the hospital’s information system, the palliative care team can be called on by every specialist department in the hospital. The presence of the palliative care service is documented using OPS code 8–982 (specialist complex palliative care). Cases in which this code was documented were recorded and statistically analyzed.

### Statistical analyses

The recorded frequencies of clinical characteristics, operations, medical procedures and contact with the hospital palliative care team were presented in absolute and percentage figures. Calculations were carried out for the complete group of all cancer patients who died (C00-C96) and also for deceased patients with solid tumors (C00-C80) as well as for patients with hematological malignancies (C81-C96). We also separated cancer patients with chemotherapy at the end of life and performed a stratified analysis according to sepsis status and tumor entity (C00-C96). Sepsis was classified according to the ICD-10 codes B37.7, A39.2, A39.4, A40, A41, and R57.2.

Differences between the sub-groups were tested. Continuous data were tested for normal distribution using the Kolmogorov–Smirnov test, and were analyzed with the unpaired t-test if normally distributed. If a normal distribution was not present, the nonparametric Wilcoxon–Mann–Whitney test was used. The chi-squared test was used for categorical data, and in cases of low frequency, Fisher’s exact test with cell count less than five was used. The significance level was set at *p* < 0.05. To take into account the global increase in the likelihood of alpha error (type 1 error) with multiple testing in the same sample, the alpha level was corrected using the Holm–Bonferroni procedure. Using multivariate logistic regression, the influences of age (< 60 years (1) vs. ≥ 60 years (0)), gender (men (1) vs. women (0)), the time interval since first diagnosis of cancer (< 6 months (1) vs. ≥ 6 months (0)), and tumor entity (hematological malignancies (1) vs. solid (0) tumors) were investigated relative to the implementation of resuscitation measures, surgery of whatever sort, renal replacement procedures, and blood transfusions 1 month and 1 week before the death of the cancer patient. Odds ratios (OR) and 95% confidence intervals (CI) were calculated. The two-sided Wald statistic was used to test significance. Log likelihood (–2LL) and Nagelkerke’s pseudo-*R*^2^ coefficient were used to evaluate the quality of the multivariate model. The analyses were carried out using the IBM SPSS Statistics program, version 23.

### Ethics approval

The study was submitted to the University of Munich’s ethics committee and obtained ethics approval (ref. no. 443–15 UE). Due to the anonymized analysis of the data, consultation with the ethics committee, albeit it was carried out, was not an absolute requirement.

## Results

**Fig 1:**
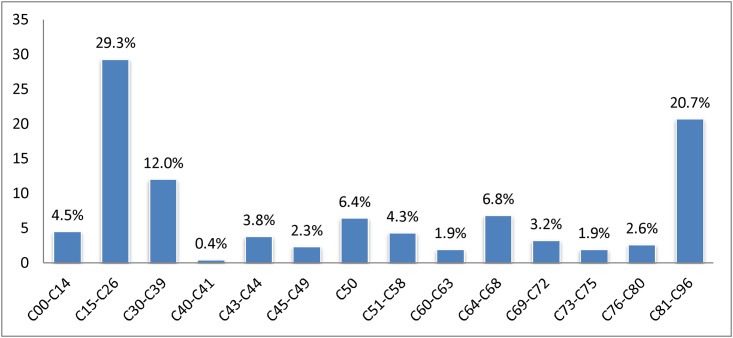
The distribution of the tumor entity of deceased cancer patients (N = 532). C00-C14: Malignant neoplasms of lip, oral cavity and pharynx, C15-C26: Malignant neoplasms of digestive organs, C30-C39: Malignant neoplasms of respiratory and intrathoracic organs, C40-C41: Malignant neoplasms of bone and articular cartilage, C43-C44, Melanoma and other malignant neoplasms of skin, C45-C49: Malignant neoplasms of mesothelial and soft tissue, C50: Malignant neoplasms of breast, C51-C58: Malignant neoplasms of female genital organs, C60-C63: Malignant neoplasms of male genital organs, C64-C68: Malignant neoplasms of urinary tract, C69-C72: Malignant neoplasms of eye, brain and other parts of central nervous system, C73-C75: Malignant neoplasms of thyroid and other endocrine glands, C76-C80: Malignant neoplasms of ill-defined, other secondary and unspecified sites, C81-C96: Malignant neoplasms of lymphoid, hematopoietic and related tissue.

A total of 532/1222 patients (43.5%) died of cancer during 2014. Of these patients, 422/532 (79.3%) had a solid tumor and 110/532 (20.3%) a malignant neoplasm of lymphoid, hematopoietic and related tissue. Malignant tumors of the gastrointestinal tract (C15-C26) were most frequent (29.3%). One in ten patients died of a malignant neoplasm of the respiratory tract (C30-C39) (Fig 1).

There were slightly more males than females among the cases of death. The mean age at death was 66.8 years. In one-third of the patients, the first diagnosis of cancer had been less than 6 months ago. In patients with solid malignant tumors, a secondary malignant neoplasm was documented in at least 62.1%. A bone marrow transplant was performed in one-fifth of patients with hematological malignancy (Table 1).

**Table 1:** Characteristics of deceased cancer patients.

	All cancer patients		Solid tumor		Hematological malignancy		
	N = 532		N = 422		N = 110		
	n	(%)	n	(%)	n	(%)	*P*
**Gender**							
Female	219	(41.2)	169	(40.0)	50	(45.5)	
Male	313	(58.8)	253	(60.0)	60	(54.5)	0.305
**Age**							
Mean (SD)	66.8	(14.8)	67.0	(14.0)	65.8	(17.8)	0.435
< 40 years	25	(4.7)	17	(4.0)	8	(7.3)	0.152
40–59 years	104	(19.5)	88	(20.9)	16	(14.5)	0.137
60–79 years	326	(61.3)	257	(60.9)	69	(62.7)	0.726
≥ 80 years	77	(14.5)	60	(14.2)	17	(15.5)	0.743
Duration between diagnosis and cancer death*							
< 6 months	173	(33.6)	140	(34.5)	33	(30.5)	0.527
6–12 months	73	(14.2)	52	(12.8)	21	(19.5)	0.066
≥ 12 months	268	(52.2)	214	(52.7)	54	(50.0)	0.762
**Malignant solid tumors (ICD-10; C00-C80)**							
Secondary malignant neoplasm (ICD-10; C77 or C78 or C79)			262	(62.1)			
- Lymph nodes (ICD-10; C77)			101	(23.9)			
- Respiratory and digestive organs (ICD-10; C78)			195	(46.2)			
- Other and unspecified sites (ICD-10; C79)			145	(34.4)			
- Only lymph nodes (ICD-10; C77)			13	(3.1)			
- Only respiratory and digestive organs (ICD-10; C78)			72	(17.1)			
- Other and unspecified sites (C79)			37	(8.8)			
- ICD-10; C77 and C78			32	(7.6)			
- ICD-10; C77 and C79			17	(4.0)			
- ICD-10; C78 and C79			52	(12.3)			
- ICD-10; C77 and C78 and C79			39	(9.2)			
**Malignant neoplasms of lymphoid, hematopoietic and related tissue (ICD-10; C81-C96)**							
Bone morrow transplant status (ICD-10; Z94.80 or Z94.81)					22	(20.0)	
Not having achieved remission#					61	(55.5)	
- Malignant immunproliferative diseases, certain B-cell lymphomas (C88.00, C88.20, C88.30, C88.40, C88.70)					0	(0.0)	
- Multiple myeloma and malignant plasma cell neoplasms (C90.0, C90.10, C90.20, C90.30)					18	(16.4)	
- Lymphoid leukemia (C91.00, C91.10, C91.30, C91.40, C91.50, C91.60, C91.70, C91.80, C91.90)					11	(10.0)	
- Myeloid leukemia (C92.00, C92.10, C92.20, C92.30, C92.40, C92.50, C92.60, C92.70, C92.80, C92.90)					30	(27.3)	
- Monocytic leukemia (C93.00, C93.30, C93.70, C93.90)					1	(0.9)	
- Other leukemias of specific cell types (C94.00, C94.20, C94.30, C94.40, C94.60, C94.70, C94.8)					2	(1.8)	
- Leukemia of unspecified cell type (C95.00, C95.10, C95.70, C95.8, C95.90)					1	(0.9)	

* N = 514 all cancer patients; n = 406 patients with solid tumors, n = 108 patients with hematological malignancies.

^#^ ICD-10; C88.00, C88.20, C88.30, C88.40, C88.70, C88.90, C90.0, C90.10, C90.20, C90.30, C91.00, C91.10, C91.30, C91.40, C91.50, C91.60, C91.70, C91.80, C91.90, C92.00, C92.10, C92.20, C92.30, C92.40, C92.50, C92.60, C92.70, C92.80, C92.90, C93.00, C93.30, C93.70, C93.90, C94.00, C94.20, C94.30, C94.40, C94.60, C94.70, C94.8, C95.00, C95.10, C95.70, C95.8, C95.90.

**Fig 2:**
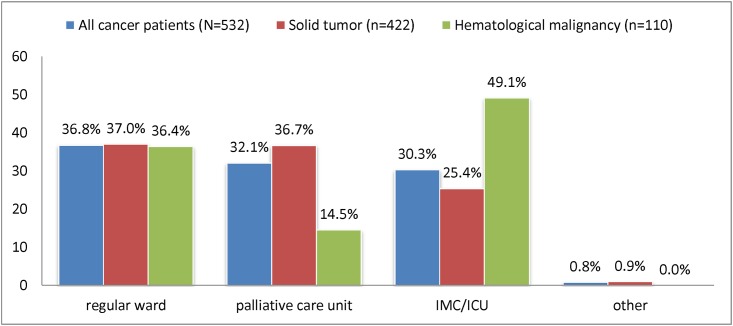
Place of death in hospital for cancer patients. IMC = Intermediate Care Unit; ICU = Intensive Care Unit.

Places of death in the hospital were distributed as follows (all cancer patients, N = 532): regular ward, n = 196 (36.8%); palliative care unit, n = 171 (32.1%); intermediate care or intensive care unit, n = 161 (30.3%); and other, n = 4 (0.8%). Compared to patients with solid tumors, patients with hematological malignancies died in an intermediate care or intensive care unit more often (25.4% vs. 49.1%, *p* = 0.001), and less often in a palliative care unit (36.7% vs. 14.5%, *p* = 0.001) (Fig 2).

Chemotherapy had been administered within the last week of life in 41/532 cancer patients (7.7%) and within the last 30 days of life in 204/532 patients (38.3%). Approximately every 5^th^ patient with a malignant tumor of the lymphatic or hematopoietic tissue and every 20^th^ patient with a solid tumor received chemotherapy within the last week. In addition, 77/110 patients with hematological malignancy (70.0%) and 127/422 patients with a solid tumor (30.1%) received tumor-specific therapy during the last month. Only a small percentage of patients received radiotherapy during the last week of life (2.6%) or last month of life (6.4%). One in ten cancer patients underwent resuscitation at the end of life. The proportions were 8.5% (last week) and 10.5% (last month). In comparison with patients with solid tumors, patients with hematological malignancies were more likely to receive blood transfusions (18.5% vs. 31.8%; *p* = 0.002), platelet transfusions (5.0% vs. 19.1%; *p* = 0.001) and renal replacement therapy (9.7% vs. 20.9%; *p* = 0.001) during their last week of life. The prevalence figures for these procedures in the last month of life were 32.7% vs. 65.5% (*p* = 0.001), 9.2% vs. 48.2% (*p* = 0.001) and 13.0% vs. 31.8% (*p* = 0.001), respectively. With regard to diagnostic measures at the end of life (last week/month), trachea-bronchoscopy procedures were carried out in 4.5%/9.0%, endoscopy of the upper GI tract in 7.7%/13.5%, magnetic resonance imaging (MRI) in 2.8%/10.5% and computed tomography (CT) in 33.8%/60.9% (Tables 2 and 3).

**Table 2:** Frequencies of medical procedures in cancer patients with malignant solid tumors compared to non-solid tumors in the last week of life.

	All cancer patients		Solid tumor		Hematological malignancy		
	N = 532		N = 422		N = 110		
	n	(%)	n	(%)	n	(%)	*P*
**Chemotherapy**	41	7.7	21	5.0	20	18.2	0.001
**Radiotherapy**	14	2.6	11	2.6	3	2.7	0.944
**Resuscitation**	45	8.5	34	8.1	11	10.0	0.514
**Surgery**	81	15.2	67	15.9	14	12.7	0.413
**Tracheotomy**	3	0.6	2	0.5	1	0.9	0.502
**PEG tube placement**	2	0.4	2	0.5	0	0.0	-
**Pleural puncture**	18	3.4	13	3.1	5	4.5	0.552
**Ascites puncture**	5	0.9	4	0.9	1	0.9	1.000
**Extracorporeal membrane oxygenation**	1	0.2	0	0.0	1	0.9	-
**Renal replacement therapy**	64	12.0	41	9.7	23	20.9	0.001
**Blood cell transfusion**	113	21.2	78	18.5	35	31.8	0.002
**Erythrocyte transfusion**	92	17.3	71	16.8	21	19.1	0.576
**Platelet transfusion**	42	7.9	21	5.0	21	19.1	0.001
**Blood plasma transfusion**	63	11.8	50	11.8	13	11.8	1.000
**Tracheo-bronchoscopy**	24	4.5	16	3.8	8	7.3	0.117
**Endoscopy of upper GI tract**	41	7.7	38	9.0	3	2.7	0.027
**Computed tomography (CT)**	180	33.8	141	33.4	39	35.5	0.687
**Magnetic Resonance Imaging (MRI)**	15	2.8	11	2.6	4	3.6	0.525

**Table 3:** Frequencies of medical procedures in cancer patients with malignant solid tumors compared to non-solid tumors in the last month of life.

	All cancer patients		Solid tumor		Hematological malignancy		
	N = 532		N = 422		N = 110		
	n	(%)	n	(%)	n	(%)	*P*
**Chemotherapy**	204	38.3	127	30.1	77	70.0	0.001
**Radiotherapy**	34	6.4	28	6.6	6	5.5	0.652
**Resuscitation**	56	10.5	43	10.2	13	11.8	0.620
**Surgery**	165	31.0	134	31.8	31	28.2	0.471
**Tracheotomy**	18	3.4	12	2.8	6	5.5	0.177
**PEG tube placement**	5	0.9	4	0.9	1	0.9	0.970
**Pleural puncture**	22	4.1	14	3.3	8	7.3	0.064
**Ascites puncture**	32	6.0	29	6.9	3	2.7	0.103
**Extracorporeal membrane oxygenation**	6	1.1	2	0.5	4	3.6	0.019
**Renal replacement therapy**	90	16.9	55	13.0	35	31.8	0.001
**Blood cell transfusion**	210	39.5	138	32.7	72	65.5	0.001
**Erythrocyte transfusion**	194	36.5	130	30.8	64	58.2	0.001
**Platelet transfusion**	92	17.3	39	9.2	53	48.2	0.001
**Blood plasma transfusion**	100	18.8	72	17.1	28	25.5	0.045
**Tracheo-bronchoscopy**	48	9.0	29	6.9	19	17.3	0.001
**Endoscopy of upper GI tract**	72	13.5	64	15.2	8	7.3	0.031
**Computed tomography (CT)**	324	60.9	246	58.3	78	70.9	0.016
**Magnetic Resonance Imaging (MRI)**	56	10.5	44	10.4	12	10.9	0.883

Patients with malignant neoplasms of respiratory and intrathoracic organs (C30-C39) were most likely to receive chemotherapy in the last 7 days compared to patients with other malignant solid tumors. In patients with ICD-10 codes C00-C14, C15-C26, C45-C49 and C51-C58, surgery was most frequently performed during the last 30 days. Patients with neoplasms of lip, oral cavity and pharynx received tracheotomy in 12.5% of cases in the last month and in 4.2% of cases in the last week. More than one-fifth of all patients with tumor of the urinary tract were treated with renal replacement therapy (Tables 4 and 5).

**Table 4:** Cancer patients with malignant solid tumors—frequencies of medical procedures in the last week of life stratified by tumor entity.

	Patient with malignant solid tumors, N = 422												
Medical procedures in the last 7 days	C00-C14	C15-C26	C30-C39	C40-C41	C43-C44	C45-C49	C50	C51-C58	C60-C63	C64-C68	C69-C72	C73-C75	C76-C80
	n = 24	n = 156	n = 64	n = 2	n = 20	n = 12	n = 34	n = 23	n = 10	n = 36	n = 17	n = 10	n = 14
	(%)	(%)	(%)	(%)	(%)	(%)	(%)	(%)	(%)	(%)	(%)	(%)	(%)
**Chemotherapy**	8.3	2.6	12.5	0.0	5.0	8.3	0.0	4.3	0.0	5.6	5.9	0.0	7.1
**Radiotherapy**	4.2	0.6	12.5	0.0	0.0	0.0	0.0	0.0	0.0	0.0	0.0	10.0	0.0
**Resuscitation**	25.0	5.1	7.8	0.0	0.0	0.0	11.8	8.7	10.0	13.9	5.9	20.0	0.0
**Surgery**	16.7	21.8	4.7	0.0	5.0	25.0	2.9	21.7	20.0	19.4	11.8	20.0	21.4
**Tracheotomy**	4.2	0.6	0.0	0.0	0.0	0.0	0.0	0.0	0.0	0.0	0.0	0.0	0.0
**PEG tube placement**	0.0	0.6	0.0	0.0	0.0	0.0	0.0	0.0	0.0	0.0	0.0	10.0	0.0
**Pleural puncture**	8.3	3.2	6.3	0.0	5.0	0.0	2.9	0.0	0.0	0.0	0.0	0.0	0.0
**Ascites puncture**	0.0	1.3	1.6	0.0	0.0	0.0	0.0	0.0	0.0	0.0	0.0	0.0	0.0
**Extracorporeal membrane oxygenation**	0.0	0.0	0.0	0.0	0.0	0.0	0.0	0.0	0.0	0.0	0.0	0.0	0.0
**Renal replacement therapy**	8.3	13.5	6.3	0.0	5.0	8.3	2.9	0.0	20.0	22.2	0.0	0.0	7.1
**Blood cell transfusion**	20.8	17.9	20.3	0.0	15.0	41.7	11.8	4.3	20.0	22.2	5.9	20.0	42.9
**Erythrocyte transfusion**	20.8	17.3	17.2	0.0	10.0	41.7	8.8	4.3	20.0	16.7	5.9	20.0	42.9
**Platelet transfusion**	4.2	5.8	3.1	0.0	5.0	8.3	5.9	4.3	10.0	8.3	0.0	0.0	0.0
**Blood plasma transfusion**	4.2	17.9	4.7	0.0	10.0	33.3	5.9	0.0	10.0	13.9	5.9	10.0	14.3
**Tracheo-bronchoscopy**	8.3	3.2	10.9	0.0	5.0	0.0	2.9	0.0	0.0	0.0	0.0	0.0	0.0
**Endoscopy of upper GI tract**	12.5	13.5	1.6	0.0	5.0	0.0	8.8	4.3	10.0	11.1	5.9	10.0	7.1
**Computed tomography (CT)**	25.0	31.4	37.5	0.0	20.0	58.3	29.4	21.7	40.0	50.0	17.6	40.0	50.0
**Magnetic Resonance Imaging (MRI)**	0.0	0.6	6.3	0.0	5.0	0.0	2.9	4.3	0.0	2.8	5.9	10.0	0.0

**Table 5:** Cancer patients with malignant solid tumors—frequencies of medical procedures in the last month of life stratified by tumor entity.

	Patient with malignant solid tumors, N = 422												
Medical procedures in the last 30 days	C00-C14	C15-C26	C30-C39	C40-C41	C43-C44	C45-C49	C50	C51-C58	C60-C63	C64-C68	C69-C72	C73-C75	C76-C80
	n = 24	n = 156	n = 64	n = 2	n = 20	n = 12	n = 34	n = 23	n = 10	n = 36	n = 17	n = 10	n = 14
	(%)	(%)	(%)	(%)	(%)	(%)	(%)	(%)	(%)	(%)	(%)	(%)	(%)
**Chemotherapy**	16.7	28.8	46.9	0.0	15.0	33.3	38.2	34.8	10.0	19.4	23.5	50.0	21.4
**Radiotherapy**	8.3	1.3	20.3	0.0	15.0	0.0	5.9	0.0	0.0	5.6	11.8	20.0	0.0
**Resuscitation**	25.0	7.7	12.5	0.0	0.0	0.0	11.8	8.7	10.0	16.7	11.8	20.0	0.0
**Surgery**	37.5	41.0	14.1	0.0	10.0	50.0	2.9	39.1	30.0	50.0	29.2	30.0	35.7
**Tracheotomy**	12.5	2.6	1.6	0.0	0.0	8.3	0.0	0.0	0.0	5.6	5.9	0.0	0.0
**PEG tube placement**	4.2	1.3	0.0	0.0	0.0	0.0	0.0	0.0	0.0	0.0	0.0	10.0	0.0
**Pleural puncture**	8.3	3.2	7.8	0.0	5.0	0.0	2.9	0.0	0.0	0.0	0.0	0.0	0.0
**Ascites puncture**	0.0	13.5	1.6	0.0	0.0	0.0	11.8	8.7	0.0	2.1	0.0	0.0	0.0
**Extracorporeal membrane oxygenation**	0.0	0.0	1.6	0.0	0.0	0.0	0.0	0.0	0.0	0.0	5.9	0.0	0.0
**Renal replacement therapy**	8.3	17.3	9.4	0.0	5.0	33.3	2.9	0.0	20.0	27.8	5.9	0.0	7.1
**Blood cell transfusion**	33.3	37.2	31.3	0.0	15.0	50.0	14.7	26.1	40.0	36.1	29.4	30.0	50.0
**Erythrocyte transfusion**	29.2	35.3	28.1	0.0	15.0	50.0	11.8	26.1	40.0	33.3	29.4	30.0	50.0
**Platelet transfusion**	4.2	12.8	7.8	0.0	5.0	8.3	5.9	4.3	10.0	11.1	5.9	10.0	7.1
**Blood plasma transfusion**	12.5	26.3	10.9	0.0	10.0	33.3	5.9	0.0	10.0	19.4	5.9	10.0	21.4
**Tracheo-bronchoscopy**	8.3	5.8	20.3	0.0	5.0	0.0	2.9	0.0	0.0	0.0	5.9	20.0	0.0
**Endoscopy of upper GI tract**	20.8	23.7	1.6	0.0	5.0	8.3	14.7	8.7	20.0	16.7	5.9	20.0	7.1
**Computed tomography (CT)**	41.7	55.1	62.5	0.0	40.0	75.0	52.9	56.5	50.0	72.2	82.4	60.0	78.6
**Magnetic Resonance Imaging (MRI)**	4.2	6.4	10.9	0.0	10.0	16.7	5.9	4.3	0.0	11.1	47.1	20.0	35.7

Patients with hematological malignancies were more likely to develop septic complications after chemotherapy compared to patients with malignant solid tumors. The corresponding frequencies were 68.8% versus 31.3% (last 7 days) and 64.9% versus 35.1% (last 30 days), respectively (Tables 6 and 7).

**Table 6:** Cancer patients with chemotherapy treatment in the last week of life—frequencies of sepsis stratified by tumor entity.

	Chemotherapy within the last 7 days		No sepsis		Sepsis*		
	N = 41		N = 25		N = 16		
	n	(%)	n	(%)	n	(%)	*P*
**Solid tumors (ICD-10; C00-C80)**	21	51.2	16	64.0	5	31.3	0.041
- lip, oral cavity, pharynx (C00-C14)	2	4.9	2	8.0	0	0.0	-
- digestive organs (C15-C26)	4	9.8	2	8.0	2	12.5	0.637
- respiratory and intrathoracic organs (C30-C39)	8	19.5	7	28.0	1	6.3	0.086
- bone and articular cartilage (C40-C41)	0	0.0	0	0.0	0	0.0	-
- skin (C43-C44)	1	2.4	1	4.0	0	0.0	-
- mesothelial and soft tissue (C45-C49)	1	2.4	1	4.0	0	0.0	-
- breast (C50)	0	0.0	0	0.0	0	0.0	-
- female genital organs (C51-C58)	3	1.9	2	2.8	1	1.1	0.587
- male genital organs (C60-C63)	3	1.9	1	1.4	2	2.2	0.998
- urinary tract (C64-C68)	14	8.7	3	4.2	11	12.4	0.092
- eye, brain and other parts of CNS (C69-C72)	4	2.5	3	4.2	1	1.1	0.326
- thyroid and other endocrine glands (C73-C75)	1	0.6	0	0.0	1	1.1	-
- other secondary and unspecified sites (C76-C80)	3	1.9	1	1.4	2	2.2	0.998
**Lymphoid, hematopoietic and related tissue (ICD-10;C81-C96)**	20	48.8	9	36.0	11	68.8	0.041
- bone marrow transplant status	4	9.8	0	0.0	4	25.0	-
- not having achieved remission	12	29.3	5	20.0	7	43.8	0.103

* ICD-10: B37.7, A39.2, A39.4, A40, A41, R57.2; CNS = central nervous system

**Table 7:** Cancer patients with chemotherapy treatment in the last month of life—frequencies of sepsis stratified by tumor entity.

	Chemotherapy within the last 30 days		No sepsis		Sepsis*		
	N = 204		N = 147		N = 57		
	n	(%)	n	(%)	n	(%)	*P*
**Solid tumors (C00-C80)**	127	62.3	107	72.8	20	35.1	0.001
- lip, oral cavity, pharynx (C00-C14)	4	2.0	4	2.7	0	0.0	-
- digestive organs (C15-C26)	45	22.1	34	23.1	11	19.3	0.554
- respiratory and intrathoracic organs (C30-C39)	30	14.7	29	19.7	1	1.8	0.001
- bone and articular cartilage (C40-C41)	0	0.0	0	0.0	0	0.0	-
- skin (C43-C44)	3	1.5	3	2.0	0	0.0	-
- mesothelial and soft tissue (C45-C49)	4	2.0	4	2.7	0	0.0	-
- breast (C50)	13	6.4	11	7.5	2	3.5	0.523
- female genital organs (C51-C58)	8	3.9	8	5.4	0	0.0	-
- male genital organs (C60-C63)	1	0.5	0	0.0	1	1.8	-
- urinary tract (C64-C68)	7	3.4	6	4.1	1	1.8	0.676
- eye, brain and other parts of CNS (C69-C72)	4	2.0	3	2.0	1	1.8	1.000
- thyroid and other endocrine glands (C73-C75)	5	2.5	3	2.0	2	3.5	0.620
- other secondary and unspecified sites (C76-C80)	3	1.5	2	1.4	1	1.8	1.000
**Lymphoid, hematopoietic and related tissue (C81-C96)**	77	37.7	40	27.2	37	64.9	0.001
- bone marrow transplant status	17	8.3	3	2.0	14	24.6	0.001
- not having achieved remission	45	22.1	21	14.3	24	42.1	0.001

* ICD-10: B37.7, A39.2, A39.4, A40, A41, R57.2; CNS = central nervous system

Tumor entity and time of first diagnosis were significantly associated with therapeutic measures being administered at the end of life (resuscitation, surgery, renal replacement procedures, blood cell transfusions). For example, the probability of receiving renal replacement procedures in the last phase of life (week/month) among patients with hematological malignancies was increased in comparison with patients with solid malignant tumors (OR 2.65; 95% CI, 1.49 to 4.70; *p* = 0.001 / OR 3.47; 95% CI, 2.09 to 5.75; *p* = 0.001). A markedly increased likelihood of transfusion of blood products was also observed (OR 2.21; 95% CI, 1.36 to 3.58; *p* = 0.001 / OR 4.48; 95% CI, 2.83 to 7.12; *p* = 0.001) (Table 8).

**Table 8:** Factors associated with aggressive cancer care at the end of life.

	**Resuscitation**			**Surgery**		
	**OR**	**95% CI**	***P***	**OR**	**95% CI**	***P***
**Last 7 days**						
Gender: male (1) vs. female (0)	0.73	0.39–1.36	0.320	0.72	0.44–1.17	0.184
Age: < 60 (1) vs. ≥ 60 (0) years	0.68	0.31–1.53	0.352	1.06	0.60–1.85	0.846
Initial diagnosis: < 6 (1) vs. ≥ 6 (0) months	2.13	1.14–3.95	0.017	2.00	1.23–3.25	0.005
Cancer type: hematological malignancy (1) vs. solid (0) tumor	1.27	0.62–2.61	0.523	0.77	0.41–1.44	0.415
*Goodness of fit (– 2 log likelihood/Nagelkerke’s R*^*2*^*)*	*300*.*482/0*.*033*			*444*.*225/0*.*031*		
**Last 30 days**						
Gender: male (1) vs. female (0)	0.70	0.40–1.23	0.216	1.16	0.79–1.71	0.447
Age: < 60 (1) vs. ≥ 60 (0) years	0.96	0.49–1.87	0.904	1.24	0.80–1.92	0.346
Initial diagnosis: < 6 (1) vs. ≥ 6 (0) months	1.83	1.04–3.23	0.036	2.40	1.63–3.53	0.001
Cancer type: hematological malignancy (1) vs. solid (0) tumor	1.19	0.61–2.29	0.630	0.88	0.55–1.41	0.584
*Goodness of fit (– 2 log likelihood/Nagelkerke’s R*^*2*^*)*	*352*.*199/0*.*022*			*637*.*680/0*.*055*		
	**Renal replacement therapy**			**Blood cell transfusion**		
	**OR**	**95% CI**	***P***	**OR**	**95% CI**	***P***
**Last 7 days**						
Gender: male (1) vs. female (0)	1.51	0.85–2.67	0.157	0.99	0.64–1.54	0.982
Age: < 60 (1) vs. ≥ 60 (0) years	0.81	0.41–1.59	0.535	1.42	0.87–2.32	0.165
Initial diagnosis: < 6 (1) vs. ≥ 6 (0) months	2.19	1.28–3.76	0.004	2.48	1.64–3.84	0.001
Cancer type: hematological malignancy (1) vs. solid (0) tumor	2.65	1.49–4.70	0.001	2.21	1.36–3.58	0.001
*Goodness of fit (– 2 log likelihood/Nagelkerke’s R*^*2*^*)*	*370*.*489/0*.*073*			*524*.*063/0*.*074*		
**Last 30 days**						
Gender: male (1) vs. female (0)	1.93	1.16–3.22	0.011	1.17	0.80–1.71	0.434
Age: < 60 (1) vs. ≥ 60 (0) years	1.14	0.65–1.99	0.653	1.83	1.18–2.82	0.007
Initial diagnosis: < 6 (1) vs. ≥ 6 (0) months	1.97	1.22–3.18	0.006	2.63	1.77–3.91	0.001
Cancer type: hematological malignancy (1) vs. solid (0) tumor	3.47	2.09–5.75	0.001	4.48	2.83–7.12	0.001
*Goodness of fit (– 2 log likelihood/Nagelkerke’s R*^*2*^*)*	*449*.*641/0*.*104*			*646*.*195/0*.*161*		

**Fig 3:**
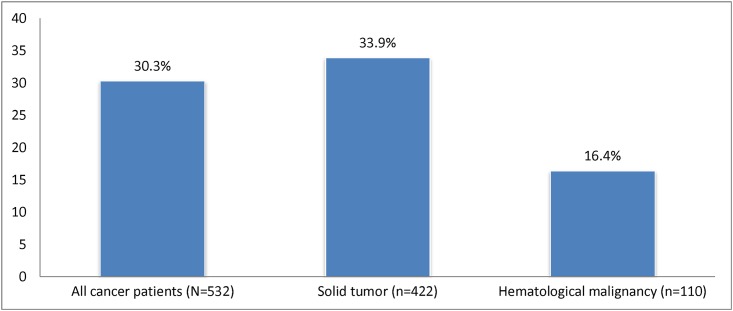
Frequency of patients’ contact with the hospital palliative care team.

Only 161/532 cancer patients (30.3%) had been in contact with the palliative care team before death. Among patients with a solid tumor, one in three (143/422) received palliative care (33.9%). Contact with the palliative care team had only been established in 18/110 patients with a hematological malignancy (16.4%), (Fig 3).

In 87/161 cases (54.0%), the inpatient hospital palliative care team was contacted within the last week of the patient’s life. In only 6.2% of the cases, the interval between first contact and death was longer than 20 days.

The duration of care of the hospital palliative care team was most often between 1 to 3 days (40.4%). One in five patients (34/161) received a period of care for more than 7 days. The length of care did not differ between cancer patients with solid tumors and patients with hematological malignancies (Table 9).

**Table 9:** Cancer patients, who had contact with the hospital palliative care team before death.

	All cancer patients		Solid tumor		Hematological malignancy		
	N = 161		N = 143		N = 18		
	n	%, (%)*	n	%, (%)*	n	%, (%)*	*P*
**Time between initial patient contact with the hospital palliative care team and the patient’s death**							
< 24 hours	9	5.6 (5.6)	7	4.9 (4.9)	2	11.1 (11.1)	0.265
1–2 days	28	17.4 (23.0)	25	17.5 (22.4)	3	16.7 (27.8)	1.000
3–4 days	24	14.9 (37.9)	19	13.3 (35.7)	5	27.8 (55.6)	0.151
5–7 days	26	16.1 (54.0)	23	16.1 (51.8)	3	16.7 (72.3)	1.000
8–13 days	40	24.9 (78.9)	37	25.8 (77.6)	3	16.7 (89.0)	0.565
14–20 days	24	14.9 (93.8)	23	16.1 (93.7)	1	5.5 (94.5)	0.479
≥ 21 days	10	6.2 (100)	9	6.3 (100)	1	5.5 (100)	1.000
**Duration of care of the hospital palliative care team**							
< 24 hours	19	11.8 (11.8)	17	11.9 (11.9)	2	11.1 (11.1)	1.000
1–3 days	65	40.4 (52.2)	57	39.9 (51.8)	8	44.4 (55.5)	0.709
4–7 days	43	26.7 (78.9)	38	26.6 (78.4)	5	27.8 (83.3)	0.913
8–13 days	27	16.8 (95.7)	24	16.8 (95.2)	3	16.7 (100)	1.000
≥ 14 days	7	4.3 (100)	7	4.8 (100)	0	0.0 (100)	-

* Cumulative percentage

## Discussion

This study describes the intensity of care provided to cancer patients who spent their final phase of life in a university hospital in Germany and died there. In general, intensive medical interventions, including life-prolonging treatments, were carried out frequently during the last phase of life. However, the intensity of medical care was associated with tumor entity. Patients with a malignant tumor of the lymphatic or hematopoietic tissue received more often intensive therapy and were more likely to die in an intensive care unit. Palliative medical expertise was only integrated into care in one third of all deceased cancer patients, often only in the very last days of life.

The study included patients in a university hospital that is required to ensure medical care at the highest medical level and to incorporate research and teaching into patient care in innovative ways. In the area of care for patients at the end of their lives, this standard also involves a risk that medical interventions may be initiated or continued even when the expected outcome is futile, and that insufficient consideration may be given for stopping or withdrawing treatment.

For patients with solid tumors, our results partially meet the intended benchmarking standards of the Earle criteria, meaning that the proportion of patients receiving chemotherapy in the last 14 days of life should be lower than 10%. Our data relate to the last 7 and not 14 days of life. We used the shorter time span as it is more likely to foresee dying within a week before death and to illustrate potential overtreatment towards the end of life more clearly.

Our reported frequencies are consistent with the international literature, in which treatment periods of two weeks or one month before death are usually reported, with period prevalence figures during the last 14 days of between 2.02% and 22.5% and during the last 30 days of between 9.0% and 43.0% [13, 14, 19–33].

The role of chemotherapy in solid tumors at the end of life is regarded increasingly critically. Its life-prolonging effect is usually slight, and there is a risk of reducing the patient’s quality of life and even lifespan. In a study including cancer patients with very advanced tumor stages who received palliative chemotherapy, Prigerson et al. [34] found that patients with good functional status (ECOG score 1) had a poorer quality of life with treatment, while in patients with a moderate or poor functional status (ECOG score 2–3), quality of life was unchanged. Otherwise, it is evident that tumor-specific drugs used in recent years are more effective with fewer side effects. A life-prolonging effect amounting to several months has been demonstrated even in patients with solid tumors such as lung, prostate, and colon carcinoma. In addition, palliative chemotherapy can also contribute to an improvement in the quality of life [35,36]. Unfortunately, the prognosis for the course of treatment cannot always be sufficiently well assessed, whether due to comorbidity, complications during current chemotherapy, or the disease progression so that the possibility of a sudden deterioration in the patient’s physical condition during treatment always has to be taken into account. It is often patients themselves who request oncological treatment, despite a hopeless disease situation and an unfavorable risk–benefit ratio. Chu et al. [9] clearly showed that patients with advanced, incurable cancer often aim to achieve the maximum gain in life expectancy while accepting a high level of toxicity. Slevin et al. [10] showed that cancer patients were willing to undergo burdensome chemotherapy even when the likelihood of a cure was only 1%.

In our study, the majority of those who received chemotherapy were patients with malignant neoplasia in the lymphatic and hematopoietic tissue. Nearly one in two patients who had received cytostatic treatment during the last 7 days of life and one in three patients who had received chemotherapy during the last 30 days of life belonged to this group. In hematological patients, for example, with acute leukemia, a curative outcome is often intended with intensive and aggressive treatment in primary therapy, and even with advanced disease, these patients can still benefit from available oncological treatments. From this point of view, the data should be interpreted cautiously with regard to overtreatment at the end of life. Especially as the Earle criteria are based on data from patients dying from lung, breast, colorectal, or other gastrointestinal cancers [12]. Therefore, it can be questioned if they are applicable to hematological malignancies. Furthermore, patients with malignant neoplasms of lymphoid, hematopoietic and related tissue often require intensive-care unit (ICU) admission due to organ failure through disease progression or treatment-related complications. The most common reasons for ICU admission in this population include sepsis and respiratory failure. Mortality in patients with hematologic malignancy admitted to ICU varies from 33% to 69% in some studies [37,38]. We observed that more than the half of these patients with chemotherapy treatment developed septic complications and died mostly in the intensive care unit. Patients with hematological malignancies were more likely than those with solid tumors to have erythrocyte transfusion, platelet transfusion, blood plasma transfusion, renal replacement therapy and extracorporeal membrane oxygenation in the last 30 days of life. In consequence, diagnostic measures were also used more widely in this patient group illustrating the strong motivation of physicians to treat the underlying disease as long as possible. Unfortunately, this disease-specific focus hindered specialist palliative care involvement as demonstrated in less contact to the hospital palliative care team and less frequent admissions and deaths on the palliative care unit. This is consistent with a meta-analysis of 16 studies from various countries demonstrating that hematology patients were more likely to die in hospital [39] and other population-based studies that reported poorer quality of end-of-life care among hematologic patients [14,40,41].

The current study showed that about one in ten cancer patients underwent resuscitation during their last month of life. Little is known about the prevalence of resuscitation in cancer patients at the end of life. In a Taiwanese study, 5-year prevalence rate of 10.5% in cancer patients was reported [42]. Young, male, unmarried individuals and patients with underlying malignant hematological conditions, or non-metastatic tumor stages, as well those with as a recent diagnosis, were predisposed to undergo resuscitation. Only 6.2% of cancer patients are able to leave the hospital after successful in-hospital resuscitation [43]. We could not assess whether any “do not resuscitate” (DNR) orders were applied to individual patients. Literature data show that a DNR order is usually only arranged and recorded in writing very shortly before death [44] and that the omission of resuscitation measures is not discussed frequently with patients [45].

Patients with malignant diseases often suffer from physical symptoms and stressful psychosocial situations—not only in very advanced situations or when they are near death, but already earlier in the disease trajectory. The need for early integration of palliative care into the treatment of patients with incurable cancer and the wide-ranging benefits is nowadays uncontroversial and well supported by evidence [15, 46–55]. Early integration of palliative care leads to reduced burden of physical and mental symptoms, fewer hospital admissions, shorter hospitalization periods, improved perception of the disease prognosis among patients, larger numbers of transfers to a hospice, longer hospice stays, and fewer aggressive treatment procedures at the end of life. In general, this leads to better quality of life of patients and families and greater acceptance of the disease situation. In addition, cost reductions in the health care sector are also a possible result [56–61]. The term “early integration” is widely used and normally seen as months to years before death without a clear timeframe. The German Palliative Care Guidelines suggest to involve palliative care at the diagnoses of the incurability of an oncological disease [18]. Others have suggested to define entity specific stages for every type of cancer [62].

In our study only one-third of the cancer patients who died received support from the palliative care team during their hospital treatment, more than half of them only in the last week of life. The time interval between first contact with the palliative service and the patient’s death was longer than 3 weeks only in a minority of patients. Early integration of palliative care in the course of treatment thus hardly took place.

In everyday clinical work, the decision to include palliative care mainly depends on the physician primarily treating the patient and the inclusion of a palliative care team does not appear expedient to all oncologists [63]. The term “palliative care” is often misunderstood as terminal care only [64]. Some physicians also believe that merely mentioning the term “palliative medicine” will deprive the patient of any hope and that the term is associated with the stigma of death. In that view, including a palliative care team in patient care would simply represent an additional burden for the patient [65]. There are also barriers to the introduction of systematic outcome measurements that would document patients’ needs and the effectiveness of palliative medical interventions [66].

What opportunities are available for integrating palliative care into in-patient oncological care structures? Conceivable approaches might be, for example: an obligatory consultation with a palliative care physician or specialist about the palliative services available, at the time when the cancer is diagnosed or when an incurable tumor stage is reached; joint visits by oncologists and palliative care specialists to the oncology ward; recognizing and registering patients with palliative care needs in a timely fashion by trained medical and nursing staff (with a palliative care representative) in every treatment setting (emergency department, outpatient oncology department, general ward, intensive-care unit); and inclusion of a palliative physician or medical/nursing palliative specialist in tumor conferences. More detailed and specific information about palliative medicine and communicative skills should also be provided during basic training for physicians and nurses, to ensure that well-informed patients are able to clarify any open questions at an early stage for the purposes of forward-looking health care planning, able to correctly assess the prognosis for the cancer, and able to express their wishes for the last phase of life. These targets should be included in the specialist societies’ guidelines and in the criteria for certification of specialist oncology centers, and they should be regularly audited. Last but not least, the financial basis and facilities for charging the corresponding costs need to be established in order to ensure that such structures become sustainable.

### Limitations

The study is based on anonymized patient data. Accordingly, there was no information available on factors that might have influenced the use of aggressive treatment procedures at the end of life, such as the patient’s general physical condition, comorbidity, tumor stage, treatment approach (curative or palliative), type of chemotherapy, complications during the course of disease, patient’s treatment preferences, and medical instruction to limit treatment (e.g., do not resuscitate, DNR). In this context, it was not possible to draw any conclusions regarding the reasons for intensified treatment measures being administered at the end of life. The study was limited to deceased cancer patients. It can be assumed that these patients were mainly at a very advanced stage of the disease at the time when the data were collected. Patients with early-stage cancers may accordingly have been underrepresented, so that early integration of the in-patient palliative service into the course of oncological treatment might be assessed too conservatively.

## Conclusions

At the end of their lives, cancer patients receive a large number of therapeutic and diagnostic procedures. Early integration of specialist palliative care to oncological treatment might help reduce the level of potential overtreatment. Sufficient structures and concepts have to be developed and implemented to improve patient care in acute hospitals.

## Supporting information

S1 Appendix STROBE statement ─ checklist ─ cross-sectional studies. (DOC)

S2 Appendix Database. (XLSX)
